# Intention to Use Mobile Easy Payment Services: Focusing on the Risk Perception of COVID-19

**DOI:** 10.3389/fpsyg.2022.878514

**Published:** 2022-05-23

**Authors:** JiWon Kim, Mincheol Kim

**Affiliations:** Department of Faculty of Data Science for Sustainable Growth, Jeju National University, Jeju City, South Korea

**Keywords:** mobile easy payment service, non-linear relationship, extended technology acceptance model, COVID-19, WarpPLS

## Abstract

Since the COVID-19 outbreak, the use of mobile easy payment services that minimize human contact has rapidly increased. Several studies have explored the relationship between the COVID-19 pandemic and the intention to use mobile easy payment services, assuming that the relationship between both variables is simply linear. However, actual complex relationships between variables cannot be fully analyzed in a linear fashion, as most relationships between variables of social phenomena are non-linear. Therefore, this study attempted to analyze the non-linear relationships between factors influencing the intention to use mobile easy payment services, especially since the COVID-19 outbreak, by applying the extended technology acceptance model (TAM2). Online and offline surveys were conducted with users who have used mobile easy payment services since the COVID-19 outbreak; 227 samples were secured for analysis. In addition, an empirical analysis was conducted using PLS-SEM to determine the linearity of relationships between variables. The results showed that subjective norms, perceived ease of use, and perceived usefulness had significant effects on the intention to use mobile easy payment services. Moreover, the COVID-19 pandemic had a significant moderating effect, also implying non-linear relationships between variables. Based on these results, the study proposes that the pandemic is a factor influencing the intention to use mobile easy payment services, and recommends that providers adopt marketing strategies, such as improving the usefulness of these services.

## Introduction

With recent developments in information and communication technology, increased smartphone penetration, and deregulation of online financial services, fintech industries related to internet banking, online payments, and electronic money, among others, are rapidly emerging as global innovations (You et al., 2018; Goldstein et al., 2019). Of all global fintech industries, the mobile payment service sector has grown most remarkably, especially with the spread of smartphones and online shopping (Park et al., 2017; Eun and Kim, 2018). A mobile easy payment service stores card information on mobile devices, such as smartphones and personal digital assistants, and pays for goods or services in lieu of cash, checks, or credit cards. It is a kind of electronic banking service (Qu and Kim, 2019). Compared with existing payment methods, this service can be used in more diverse transaction situations, such as online payments. It has significantly changed individuals’ daily lives by being a convenient, safe, and fast payment system than can be used anytime and anywhere (Putri et al., 2020). Accordingly, several domestic and foreign companies have launched various mobile easy payment services that allow users to make payments easily *via* a simple authentication process. Users have to provide their card or account information only once, that is, during the initial payment. The popularity of these services has expanded their market rapidly, and according to the American market research company Allied Market Research (2020), the global mobile easy payment service market is expected to grow from $1.912 trillion in 2020 to $12.62 trillion in 2027, at a compound annual growth rate of 30.1%. The Korea Innovation Foundation and Mobile Payment Market (2021) also predicts an increase.

COVID-19, apart from being a threat to personal health worldwide, is highly contagious. Since its detection in China’s Hubei province in 2019, the number of infected patients across the world increased rapidly, leading to its relatively quick declaration as a global pandemic (Velavan and Meyer, 2020). To prevent its spread, countries worldwide implemented strong quarantine policies at national levels, such as social distancing, national blockades, and restrictions on interpersonal contact. Preventive actions at the individual level, such as minimizing social contact, frequent hand washing, and wearing a mask, were also promoted (Hong et al., 2021).

In the context of COVID-19, contactless mobile easy payment services are becoming important as alternatives to cash and credit card payments (Bank of Korea, 2021). These services contribute to reducing the risk of COVID-19 infections owing to their remote, contactless, and convenient payment methods (Zhao and Bacao, 2021). This is especially why mobile payment services have been widely adopted as innovative financial transactions in the COVID-19 business environment, thereby solidifying their tremendous market potential (De Luna et al., 2019). Several international studies suggest that COVID-19 is one of the factors affecting the use of mobile easy payment services (Immanuel and Dewi, 2020; Daragmeh et al., 2021). Rafdinal and Senalasari (2021) analyzed the adoption of mobile payment applications during the COVID-19 pandemic, and found that users’ attitudes were influenced by the perceived usefulness and ease of use, which are variables of the Technology Acceptance Model. Zhao and Bacao’s (2021) study integrated the Unified Theory of Acceptance and Use of Technology (UTAUT) with the perceived benefits of the Mental Accounting Theory (MAT). Its empirical results showed that users’ technological and mental perceptions conjointly influenced their intention to adopt mobile payment during the COVID-19 pandemic.

Although previous studies explained how COVID-19 risk perception (C19RP) was an influencing factor in mobile easy payment services’ use intention (UI) after the onset of COVID-19, several limitations remained. For instance, the relationship between the factors with a significant effect on mobile easy payment services might not always be constantly linear. Most social phenomena exist in a non-linear relationship (Sung, 2021), which is yet to be studied. Estimating that mobile easy payment services’ UI factors exist in a linear relationship only means that the dependent variable remains insufficiently explained, and that the estimation results of such studies could be biased (Yang and Kim, 2021). Overcoming this limitation requires a novel interpretation that analyzes the non-linear relationship between the phenomena from multiple perspectives.

Therefore, to identify and analyze mobile easy payment services’ UI factors amidst the COVID-19 pandemic, this study applied the extended Technology Acceptance Model (TAM2). The study examined the use of mobile easy payment services and added a moderator variable called C19RP to verify the variables’ relationship in a non-linear manner. An empirical study on the moderating effect on UI was conducted to enhance the explanation of mobile easy payment services’ UI after the onset of COVID-19.

## Literature Review

TAM2 is a modified version of the Technology Acceptance Model (TAM) proposed by Davis (1989). Based on the Theory of Reasoned Action (TRA), TAM provides a useful theoretical framework for explaining the factors that influence people’s adoption of new technologies. It explains how the perceived ease of use and usefulness affect use intention. However, it was criticized for not taking into account external factors that could influence the technology adoption process. In response, Davis, along with Viswanath and Venkatesh, proposed TAM2 by adding external factors (subjective norm, image, etc.) that affect the information technology acceptance process (Venkatesh and Davis, 2000; Hwang and Yu, 2016; Kang, 2016).

TAM2 is being used for research in various fields, to identify behavioral intention factors. Aji et al. (2020) used it to analyze the use intention of electronic money in Indonesia. The results indicated that subjective norm had a strong positive effect on use intention. Khoa et al. (2021) used it to empirically analyze the factors influencing the intention to book accommodation services through mobile applications, and similarly determined that subjective norm was a significant factor, due to the perceived ease of use and usefulness. Hwang and Yu (2016) found that socially influential factors, such as perceived ease of use/usefulness/risk, as well as the subjective norm related to the technical characteristics of mobile easy payment, affect the active acceptance of mobile easy payment.

Although several studies have applied TAM2 for identifying the intention to use mobile easy payment services, this study aims at furthering this by applying TAM2 to study intentions in the context of a pandemic. In particular, it attempts a fresh interpretation of the intention to use mobile easy payment services by determining whether the relationship between variables is a linear or non-linear one, and analyzing the possible non-linear relationship.

## Materials and Methods

### Study Setting

A study conducted in Hungary found that the COVID-19 pandemic affected the intention to use mobile easy payment services (Daragmeh et al., 2021), where more than 80% of payments before 2019 were made in cash (before COVID-19). However, such findings are not generalizable, as in several countries, including South Korea, the utilization rate of mobile easy payment services was high even before the COVID-19 pandemic.

As of 2020, the easy payment market’s share (based on the average daily usage) was 45.7%, far more than that of general financial companies (30.5%) (Jeong, 2021). In South Korea, the usage of mobile easy payment services has significantly increased with the outbreak of COVID-19. In 2020, the mean number of mobile easy payment services users was 14.55 million per day, 4.4% higher than that in 2019, prior to COVID-19; the average cost of use per day was 449.2 billion, 41.6% higher than that in 2019 (Bank of Korea, 2021). Therefore, this study aimed at identifying the factors influencing the intention to use mobile easy payment services since the COVID-19 outbreak in South Korea, which anyway had a high utilization rate of these services.

### Establishment of a Research Model

**FIGURE 1:**
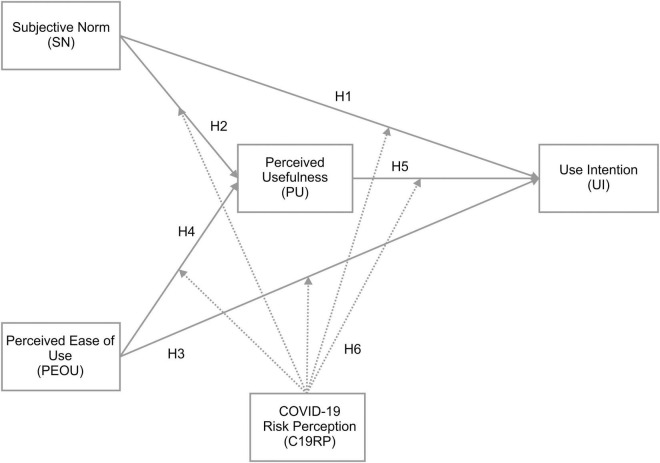
Research model.

The factors affecting mobile easy payment services’ UI after the onset of COVID-19, and the use of C19RP as a moderator variable, were examined. A research model was designed, which applied TAM2 to examine the moderating effect on UI. This included Subjective Norm (SN), one of the external factors influencing the information technology acceptance process. SN and Perceived Ease of Use (PEOU) were used as independent variables, Perceived Usefulness (PU) was used as the parameter, and UI was used as the dependent variable. The inclusion of C19RP as a moderator variable implies that it is each of the independent, parameter, and dependent variables. A research model was thereby constructed, as shown in Figure 1, to determine the kind of regulating action that occurs between the elements.

### Establishment of Research Hypothesis

#### Subjective Norm and Hypotheses About Perceived Usefulness and Use Intention

Subjective norm refers to people’s perceptions of how their behavior as users will be judged (Park and Kim, 2018). It is an important behavioral intention in the Theory of Reasoned Action and the Theory of Planned Behavior (Ajzen, 1991). The existing TAM did not consider SN. However, Venkatesh and Davis (2000) found that in actual social environments, people employed their perceived ease of use and usefulness. Hence, they proposed TAM2, which considers the factors affecting behavioral intention, along with PU. Sathye et al. (2018) explained that SN has a direct effect on the intention to use mobile payment systems, and an indirect effect through the medium of PU. Therefore, this study proposed the following hypotheses:

H1: The subjective norm has a positive effect on use intention.
H2: The subjective norm has a positive effect on perceived usefulness.

#### Hypothesis on Perceived Usefulness and Intention to Use Under Perceived Ease of Use

Perceived ease of use refers to the degree to which an individual feels comfortable to use a particular system, and is an important predictor of PU, as well as users’ intentions for accepting a particular technology (Davis, 1989). Several studies on mobile easy payment services found a positive relationship between PEOU and the intention to use mobile payment services. Cao et al. (2016), Shankar and Datta (2018), and Daragmeh et al. (2021) found that PEOU had the greatest effect on PU. Therefore, this study proposed the following hypotheses:

H3: Perceived ease of use has a positive effect on use intention.
H4: Perceived ease of use has a positive effect on perceived usefulness.

#### Hypothesis on Perceived Usefulness and Use Intention

Perceived usefulness is a variable that directly predicts users’ intention to accept a particular technology (Al-Maroof and Al-Emran, 2018), and indicates the degree to which users believe that using the system will improve their performance (Davis, 1989). Several studies on mobile easy payment services suggest that PU has a positive effect on UI (De Luna et al., 2019; Singh et al., 2020). Therefore, this study proposes the following hypothesis:

H5: Perceived usefulness has a positive effect on use intention.

#### Hypothesis on the Modulating Effect of COVID-19 Risk Perception

Risk perception is an individual’s subjective intuition for judging and recognizing a dangerous situation under specific circumstances. Early research on risk perception was primarily conducted from an economic point of view, but since the mid-19th century, many studies have been conducted in the field of consumer behavior (Bae and Jeong, 2021). Risk perception is determined by the characteristics of various national cultures, and since the COVID-19 pandemic outbreak, people’s anxieties and fears have increased. Therefore, it can be assumed that the uncertainties resulting from the spread of COVID-19 acts as individuals’ psychological risk factor, and affects their behavioral intentions (Kim, 2020).

Owing to the social distancing norms and national lockdowns implemented in some countries after the COVID-19 outbreak, consumers have been forced to use contactless-based mobile payment services, and thereby, their use has significantly increased. As people might be variously aware of the risks of COVID-19, it is difficult to find a reliable empirical study on how this affects the UI of mobile easy payment services. Therefore, this study assumes the following hypotheses to determine the influence of C19RP on perceiving the risk of COVID-19 infection, and to find out how C19RP is a modulator variable that affects the UI of mobile easy payment services:

H6-1: COVID-19 risk perception moderates the relationship between subjective norm and use intention.
H6-2: COVID-19 risk perception moderates the relationship between subjective norm and perceived usefulness.
H6-3: COVID-19 risk perception moderates the relationship between perceived ease of use and use intention.
H6-4: COVID-19 risk perception moderates the relationship between perceived ease of use and perceived usefulness.
H6-5: COVID-19 risk perception moderates the relationship between perceived usefulness and use intention.

### Research Design

As part of this study, a survey was conducted to identify the factors affecting mobile easy payment services’ UI after the onset of COVID-19. It was conducted for 2 weeks, from September 27 to October 10, 2021, using an online Google questionnaire, as well as offline face-to-face interviews.

### Sampling

Users who were experienced in using easy mobile payment services were considered eligible for the study. A total of 245 online questionnaires and 55 offline questionnaires were collected. Of these, 227 questionnaires were finally used for empirical analysis, as 65 respondents who did not use mobile easy payment services during the COVID-19 pandemic were excluded, along with eight other respondents who were unsuitable for analysis owing to omissions. This was because the recommended minimum number of samples in the Partial Least Squares-Structural Equation Modeling (PLS-SEM) model, applied in this study, was 10 times the number of connected paths, based on the variable with the most paths (Hair et al., 2021).

### Instruments

All the variables in the study were used after thoroughly reviewing the measurement items in previous studies. The composition of the questionnaire is similar to that of Daragmeh et al. (2021); some questions were modified and supplemented as per the study’s purpose. The questions were measured *via* a 5-point Likert scale, ranging from 1 (“strongly disagree”) to 5 (“strongly agree”).

### Data Collection

As of 2020, South Korea had the world’s fifth-largest digital payment market, with 88% of smartphone users having experience in using mobile easy payment services (Dmcreport, 2020; Choo, 2021). In South Korea, mobile easy payment services are a widely used payment method.

We conducted an empirical study to test the constructs that possibly influence behavioral intentions of using mobile easy payments, in light of the COVID-19 outbreak in South Korea. For this purpose, we developed a questionnaire comprised of five constructs: Subjective Norm (SN), Perceived Ease of Use (PEOU), Perceived Usefulness (PU), COVID-19 Risk Perception (C19RP), and Use Intention (UI). The study was conducted for 2 weeks, from September 27 to October 10, 2021, using online (Google Forms) and offline (face-to-face interviews) surveys. Participants were randomly selected from the South Korean adult population (over the age of 19 years) who used smartphones.

### Data Analysis

Structural equation modeling (PLS-SEM), using the partial least squares method, was used for quantitative analysis. The software used in the study was the Statistical Package for the Social Sciences (SPSS), version 28.0, and WarpPLS 7.0. The data were analyzed in two stages, beginning with the analysis of the measurement model, followed by that of the structural model.

## Results

### Sample Demographics

The respondents’ gender ratio distributed almost equally, with 45.4% male and 54.6% female. The sample’s demographic characteristics are presented in Table 1.

**TABLE 1:** Demographic characteristics.

Variable	Category	Frequency	Ratio (%)
Gender	Male	103	45.4%
	Female	124	54.6%
Age	Under 20	15	6.6%
	20–29	122	53.8%
	30–39	40	17.6%
	40–49	22	9.7%
	Over 50	28	12.3%
Education level	Middle School and below	12	5.3%
	High School	33	14.6%
	Undergraduate	166	73.1%
	Post-graduate	16	7.0%
Total	227		100%

### Factor Analysis

Prior to verifying the questionnaire’s data, an exploratory factor analysis was performed using SPSS 28.0. Principal component analysis, which extracts factors based on the overall variance, was used as a factor extraction model for the analysis. The Varimax method, which minimizes the number of variables with a high loading of each factor, was used (Kang, 2016). The factor extraction method was based on an eigenvalue of 1.0 or more, and a factor load value of ≥0.5, as per Kang (2013). Table 2 shows the results of the measurement items’ factor analysis. The questionnaire’s items were derived from five factors. The total explained variance was 74.911%. In the rotated component matrix, the factor loading values of all the measurement items were 0.5 or higher. Therefore, conceptual validity was established.

**TABLE 2:** Factor analysis result.

Variable	Category	Factor analysis				
		Factor 1	Factor 2	Factor 3	Factor 4	Factor 5
SN	SN1	0.798				
	SN2	0.793				
	SN3	0.737				
	SN4	0.832				
	SN5	0.755				
PEOU	PEOU1		0.735			
	PEOU2		0.783			
	PEOU3		0.807			
	PEOU4		0.814			
	PEOU5		0.870			
PU	PU1			0.752		
	PU2			0.713		
	PU3			0.709		
	PU4			0.643		
	PU5			0.609		
C19RP	C19RP1				0.630	
	C19RP2				0.736	
	C19RP3				0.691	
UI	UI1					0.805
	UI2					0.802
	UI3					0.830
	UI4					0.808
	UI5					0.878
Eigenvalue		3.648	4.247	3.171	1.978	4.186
Distributed explanation (%)		15.862	18.464	13.789	8.598	18.199

### Reliability and Feasibility Analysis

The Cronbach’s alpha coefficient and composite reliability (CR) values were reviewed for verifying internal consistency. Table 2 depicts the factor analysis results *via* the Cronbach’s alpha per construct.

Composite reliability is an index of reliability that examines latent variables. It should be greater than 0.7. The closer the synthetic reliability value is to one, the higher is the internal consistency. To verify this validity, average variance extracted (AVE) was used. AVE, an index for measuring convergent validity, indicates the factor’s magnitude of variance. The AVE value should lie between 0 and 1; an AVE greater than 0.5 indicates that reliability is secured. The closer the mean variance extraction value to one, the higher the questionnaire’s reliability, and convergence (Shrestha, 2021). As per Table 3, each variable is larger than the Cronbach’s α, CR, and AVE reference values. Therefore, the reliability and validity are excellent.

**TABLE 3:** Summary of the validity.

Latent variables	SN	PEOU	PU	C19RP	UI	AVE	CR	Cronbach α
SN	**0.908**					0.826	0.915	0.884
PEOU	0.353	**0.927**				0.860	0.934	0.912
PU	0.509	0.673	**0.921**			0.849	0.928	0.903
C19RP	0.467	0.618	0.727	**0.954**		0.912	0.937	0.899
UI	0.407	0.266	0.506	0.573	**0.931**	0.868	0.939	0.918

*Bold values is the square root of the AVE value for each variable.*

Discriminant validity refers to the coexistence of different concepts, with a low correlation between their measurements. The correlation coefficient of a latent variable should be a load value with an appropriate pattern. In the case of a measurement variable, it should be loaded high on the assigned factor. Discriminant validity can be verified through the coefficient of correlation value between AVE and the factors (Fornell and Larcker, 1981; Kim et al., 2011). As shown in Table 3, as a result of verifying the discriminant validity, the correlation coefficient between all constituent concepts is the AVE value, displayed on the diagonal line. In addition, it is the square of the coefficient of correlation of each factor, and the coefficient of determination; this verifies that the study’s factors have a secure discriminant validity.

### Validation of the Research Hypothesis

#### Model Validation

The PLS-SEM was used to establish this study’s research model. It is based on structural equation modeling (SEM), developed by combining path, regression, and factor analyses. SEM proposes two approaches for estimating the relationships between variables. The first is a covariance-based structural equation model (CB-SEM) for verifying the conceptual theory. This is a method of confirming (or rejecting) a set of systematic relationships. The second is the application of PLS-SEM for predicting a specific causal relationship. This is mostly used for developing a theory for maximizing predictability in exploratory research (Abbasi et al., 2021; Yoo et al., 2021).

With regard to sample size, while CB-SEM requires a minimum sample size of 200 participants, PLS-SEM requires 30–100 participants. Therefore, it can be conveniently applied to studies with small samples. While a larger sample size increases the estimation’s accuracy, CB-SEM and PLS-SEM yield similar results when the sample size comprises 250 participants or more (Shin, 2018). Considering that this study’s sample size was 227, either the CB-SEM or PLS-SEM could have been applied. The PLS-SEM model was chosen for interpreting the relationship between the two.

WarpPLS 7.0, a statistical program used for PLS-SEM analysis, was used to analyze the PLS-SEM model in this study as well. It performs both linear and non-linear analysis for determining whether the statistical assumption of the paths between the variables is linear or non-linear (WarpPLS, 2020; Sung, 2021). A non-linear relationship analysis using WarpPLS, rather than the existing PLS-SEM, yields a more accurate analysis than a linear relationship analysis method. As a result, one could observe significant non-linearity between the variables. An attempt was also made to derive an interpretation of the relationship (Kim, 2014). Therefore, this study first examined the definition of linearity, and then clarified the concept of non-linearity. First, “linear” indicates a relationship between different values. The definition of a linear relationship between two variables x and y is:


(1) a⁢x+b⁢y+c=0

where the coefficients a, b, and c are constants, independent of the variables x and y, and the relationship between the two variables defined in this manner satisfies all linear characteristics. When defining a non-linear relationship between two variables on the basis of the concept of linearity, “non-linear” refers to a relationship between two variables. It, therefore, indicates any relationship between x and y that is not expressed by Eq. 1. For example, all cases that include terms other than the linear terms regarding variables x and y, such as axn + by + c = 0 and axy = b, can be labeled as non-linear relationships (Jang and Kim, 1994).

Such a non-linear relationship analysis is primarily performed when a relationship that is otherwise difficult to explain using only linear relationship analysis appears. However, if the relationship between the variables is non-linear, and a commonly used linear relationship analysis is applied, the complex relationship between the variables cannot be analyzed completely. This results in reduced efficiency, which is a common challenge in this analysis. Therefore, when conducting research on complex social phenomena, it is necessary to examine the non-linear relationships between variables. This allows for obtaining more accurate results (Sung, 2021). Seo (2020) confirmed a U-shaped non-linear relationship between stress-levels emerging from restricted viewing due to COVID-19 and the intention of professional baseball fans. An appropriate stress-level might have a positive effect on promoting the intention to watch, by increasing the value of watching the game due to restrictions on visiting the stadium. A negative effect of the reluctance to visit a stadium because of perceiving it as an adverse situation, due to the risk of infection, was also found. Jang et al. (2021) explained that a V-shaped non-linear relationship exists between time required for commuting and the satisfaction thereof. In general, the longer the commuting time, the lower the satisfaction thereof. However, non-linear relationship analysis revealed that after a certain period, the number of household members, work experience, satisfaction with neighbors, and health variables affected satisfaction. Due to a high relative influence, it was interpreted as showing a V-shaped characteristic between commuting time and satisfaction with commuting time.

In this study, Warp 3 Stable was selected among the analysis options provided by WarpPLS, and structural model analysis was used for deriving the coefficient of determination (*R*2) value representative of the dependent variable’s explanatory power. If the value of *R*2 is 0.25 or higher, the fitness of the research model is “high.” The *R*2 for the dependent variables PU and UI was 0.59 and 0.48, respectively. Thus, Goodness of Fit (GoF) can be evaluated as “high.” It is also suggested that if the Tenenhaus GoF is 0.25 or more, as a measure of the GoF of other research models, the research model has a “medium” fit. If it is 0.36 or more, the research model has a “high” fit (Rahman et al., 2013). The GoF value of this research model is 0.670, which indicates a high fit. Further research on the model’s fitness criteria can be found in Table 4. The GoF of this research model meets the requisite criteria.

**TABLE 4:** Model fit and criteria.

Indices for model fit	Decision criteria
Average path coefficient (APC) = 0.237, *P* < 0.001	*P* < 0.05
Average R-squared (ARS) = 0.533, *P* < 0.001	*P* < 0.05
Average adjusted R-squared (AARS) = 0.523, *P* < 0.001	*P* < 0.05
Average block VIF (AVIF) = 1.464	Acceptable if ≤ 5, ideally ≤ 3.3
Average full collinearity VIF (AFVIF) = 2.165	Acceptable if ≤ 5, ideally ≤ 3.3
Tenenhaus GoF = 0.670	Small ≥ 0.1, medium ≥ 0.25, large ≥ 0.36

#### Hypothesis Test

**FIGURE 2:**
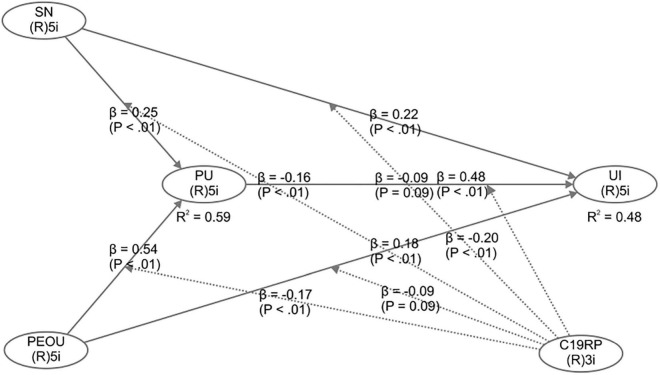
Path coefficient and model fitness level of research model.

The model was verified using PLS-SEM and the WarpPLS (7.0) software, and the linear and non-linear relationships of each linear path were analyzed. To secure the statistical significance of each path, the *p*-values of the path coefficients must be less than 0.1 (WarpPLS, 2020). Testing the hypothesis revealed that all *p* values were less than 0.1, as shown in Figure 2. This indicated the statistically significant results in all paths.

Examining the path coefficient and effect size revealed that PEOU was the path to PU, and PU was the path to UI. Statistical significance was found to be high in that order. Table 5 shows the results of the path hypotheses tests, as described above.

**TABLE 5:** Path hypotheses test.

Paths		Coefficients	Effect sizes	*P*-value	Results
H1	SN → UI	0.291	0.094	0.001***	Supported
H2	SN → PU	0.245	0.126	0.001***	Supported
H3	PEOU → UI	0.178	0.049	0.003***	Supported
H4	PEOU → PU	0.539	0.368	0.001***	Supported
H5	PU → UI	0.480	0.249	0.001***	Supported

*p < 0.1, **p < 0.05, ***p < 0.01.

The analysis of the existing structural equation model assumes that all paths are linear (Yang and Kim, 2015). We used WarpPLS software to verify whether the research hypothesis had a linear or non-linear relationship. The results of the verification showed that the paths for all hypotheses were non-linear, as shown in Table 6.

**TABLE 6:** Linear or non-linear (WARP) relationship of the paths.

Latent variables	PU	UI
SN	Warped	Warped
PEOU	Warped	Warped
PU		Warped

#### Moderation Effect Analysis

This section explains the analysis of the degree of difference in the pathways according to C19RP, which was set as a moderator variable. We reviewed the type of meaningful modulating effect of C19RP on each of the pathways, as shown in Table 7. After examining the results of hypothesis testing on the control effect of C19RP, all hypotheses with *p*-values of <0.1 were adopted. On closely reviewing the verification results, it was found that the path with the greatest difference in C19RP was from the PU to the UI. Thus, it can be concluded that the lower the group, the more sensitive the mobile easy payment service’s UI for PU. In all other pathways, lower C19RP was found to be more sensitive.

**TABLE 7:** Path hypotheses test.

Paths		Coefficients	Effect sizes	*P*-value	Results
H6-1	SN → UI	−0.089	0.008	0.087*	Supported
H6-2	SN → PU	−0.159	0.033	0.007***	Supported
H6-3	PEOU → UI	−0.087	0.026	0.093*	Supported
H6-4	PEOU → PU	−0.174	0.061	0.004***	Supported
H6-5	PU → UI	−0.197	0.052	0.001***	Supported

*p < 0.1, **p < 0.05, ***p < 0.01.

**FIGURE 3:**
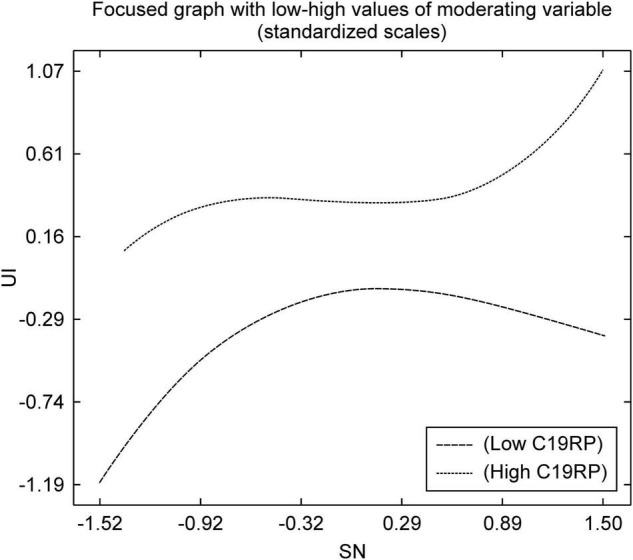
Non-linear graph of the path from SN to UI.

As shown in Figure 3, in the path from the SN to the UI, the group with a high C19RP was compared with that with a low C19RP. It was confirmed that according to SNs, the UI was high. With the implementation of social distancing, an atmosphere that encourages alternatives to face-to-face payment methods has been created. In this context, it shows that groups with high C19RP are used to mobile easy payment services. Therefore, the group with a high C19RP is more likely to use mobile easy payment services, than the group with a low C19RP, after the onset of COVID-19. This is due to social influences.

**FIGURE 4:**
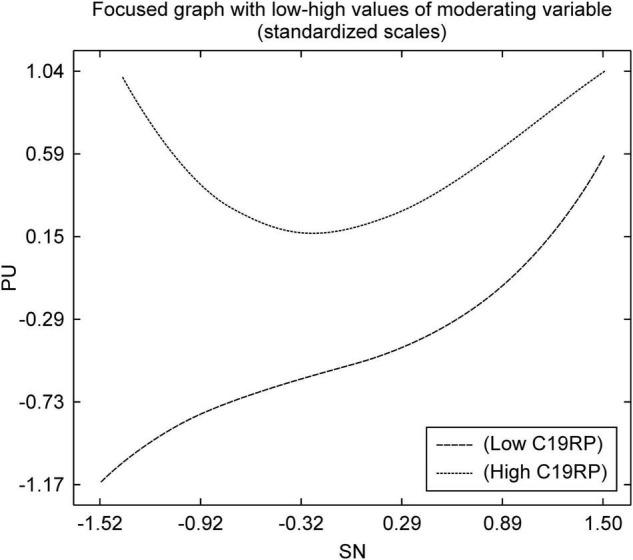
Non-linear graph of the path from SN to PU.

As shown in Figure 4, the high C19RP group, on the path from the SN to the PU, had a low C19RP. When compared with the group, it was confirmed that according to the SN, the PU was high. Although the high C19RP group showed a U-shaped non-linear relationship, when compared with the low C19RP group, perception according to the SN and PU was found to be high. This is because, in the case of a group with a high C19RP, information about COVID-19 is largely obtained from television or the internet, as well as from family and friends. Therefore, the high C19RP group is more likely to use mobile easy payment services than the low C19RP group, owing to social influences of the surrounding people and media. This might be interpreted as greater recognition of its usefulness as a means for preventing infection.

**FIGURE 5:**
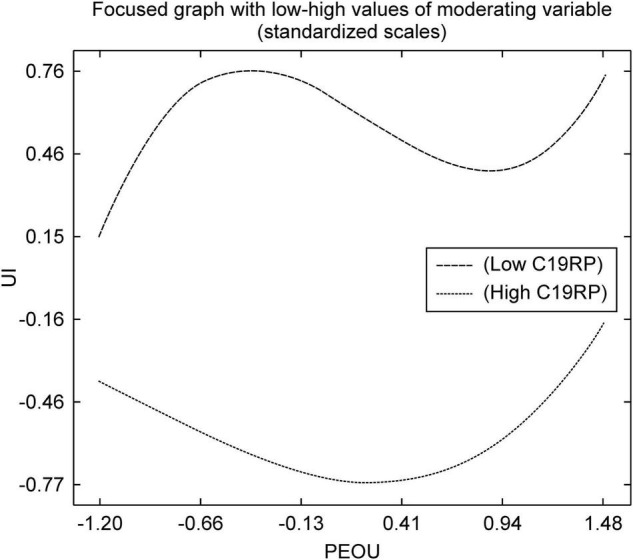
Non-linear graph of the path from PEOU to UI.

As shown in Figure 5, in the path of PEOU-to-UI, the high C19RP group had higher UI according to PEOU. A group with a high C19RP perceives that face-to-face, traditional payment methods increase the risk of contracting COVID-19, and that, to prevent infection, they must actively use contactless mobile easy payment services. Therefore, it can be interpreted that this group uses mobile easy payment services more than the group with low-risk perception. Conversely, the group with a low C19RP was less likely to perceive fear of COVID-19. Although they recognize that contactless services are easy to use and reduce the risk of infection, it can be deduced that the effect on UI is not as large as that of the group with a high C19RP.

**FIGURE 6:**
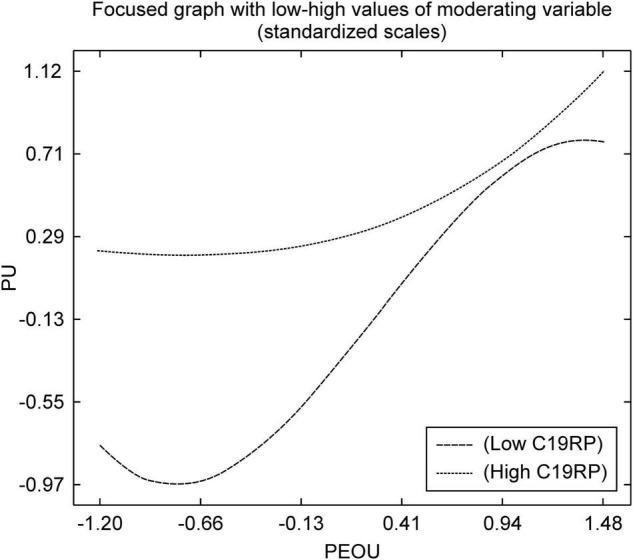
Non-linear graph of the path from PEOU to PU.

As shown in Figure 6, in the path from the PEOU to the PU, the high C19RP group had higher PU and PEOU than the group with low-risk perception. The high C19RP group perceived remote payment methods as being more capable of preventing COVID-19 infection than conventional in-person payment methods, and experienced no difficulty in using them. Therefore, it can be deduced that this group secured the usefulness of mobile easy payment services to some extent, as compared with the low C19RP group.

**FIGURE 7:**
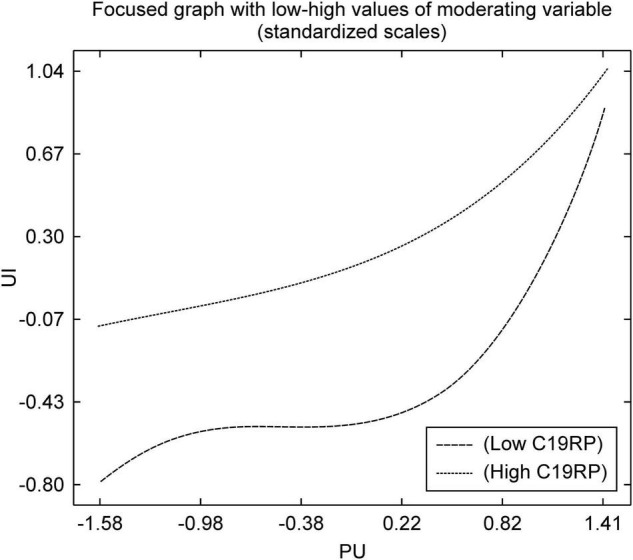
Non-linear graph of the path from PU to UI.

As shown in Figure 7, the high C19RP group had higher UI for PU than the low C19RP group. It was confirmed that the high C19RP group was already using a mobile service to some extent due to the fear of COVID-19 infection. It can be deduced that greater recognition of the usefulness of such payment services correspond to a higher positive effect on UI. Conversely, the UI of the low C19RP group was not affected significantly, even though they perceived the service as useful. Therefore, it can be deduced that in this case, the greater recognition of mobile easy payment services as useful and faster leads to a more positive effect on UI.

## Discussion

Since the outbreak of COVID-19, the utilization rate of mobile easy payment services that minimize human contact has rapidly increased. This has led to research exploring the relationship between the COVID-19 pandemic and the intention to use mobile easy payment services. Several studies assume that the relationship between the variables is simply linear. However, complex relationships between variables cannot be fully analyzed as linear, as most relationships between variables of social phenomena are in fact non-linear. Therefore, this study analyzed the non-linear relationship between factors influencing the intention to use mobile easy payment services since the COVID-19 outbreak by applying the extended technology acceptance model (TAM2).

The results of the analysis confirmed that the SN and PEOU, either directly or indirectly, had a significant effect on the intention to use mobile easy payment services. Herein, the PU plays a mediating role in the relationship between the SN/PEOU and the UI. Specifically, the greater the number of people who recommend and use mobile easy payment services, the more useful it is for the payment system, as it makes more people use the service. Moreover, it can be deduced that if a person is comfortable using mobile easy payment services, they perceive it as useful, which further motivates usage.

It was analyzed that the PU of the mobile easy payment service had a direct and significant effect on the UI. This is consistent with the findings of Kim et al. (2017), who explained that mobile services processed payments quickly and easily, and the more that consumers perceived these as useful, the more positive the intention to accept these services.

The regulatory variable C19RP had a moderating effect on all routes, meaning that the fear or anxiety of COVID-19 infection had a significant effect on the intention to use mobile easy payment services. In particular, as the path with the largest difference in C19RP, the PU was found to be more sensitive to UI in groups with lower C19RP. In addition, it can be seen that the paths from the SN to UI, SN to PU, PEOU to UI, and PEOU to PU were all more sensitive, as C19RP was lower. In other words, the group with low-risk perception of COVID-19 infection had a greater impact on the intention to use mobile easy payment services than the group with high-risk perception of COVID-19.

## Conclusion

### Research Results

Since the COVID-19 outbreak, the utilization rate of mobile payment services has sharply increased. Accordingly, many studies established COVID-19 as a factor that significantly affects the UI of mobile easy payment services. However, these studies assume that the relationship between variables is simply linear. As complex social phenomena might exist in non-linear relationships, this assumption is incorrect. To identify a mobile easy payment service’s UI in the context of COVID-19, the relationship between variables should be assumed to be both linear and non-linear.

Therefore, this study applied TAM2 on users who have used mobile easy payment services after the onset of COVID-19. This served to determine whether the SN, PEOU, and PU have any effect on UI. It also examined COVID-19 as a moderator variable for reviewing the moderating effect of risk perception. To analyze the relationship between the variables, SPSS 28.0 and WarpPLS 7.0 were used, and a summary of the specific research results is as follows:

• First, the path from the PEOU to PU is the most significant of all the routes.
• Second, using C19RP as a moderator variable, it was found that the SN, PEOU, and PU are a result of examining the effect of mobile easy payment services on the UI. Thus, it was confirmed that the paths of all the hypotheses were non-linear.
• Third, where the SN is the path to UI and the SN is PU, C19RP is a moderator variable. The path to PEOU, from the PEOU to UI, PEOU to PU, and PU were reviewed. PU has a controlling effect on the path to UI. It was found that C19RP has a significant moderating effect on the UI of mobile easy payment services.
• Fourth, when examining C19RP’s modulating effect, it was found that the group with low-risk perception of COVID-19 infection had a greater impact on the intention to use mobile easy payment services, according to the degree of COVID-19 risk perception, than the group with high-risk perception of COVID-19.
• Fifth, it was found that C19RP had the greatest moderating effect on the path from the PU to UI.

### Implications and Limitations

The study’s theoretical and practical implications are as follows. First, the WarpPLS software was used for analyzing the non-linear relationship between variables in a non-linear manner itself for arriving at a novel interpretation of the intention to use mobile easy payment services. Although many studies have analyzed UI for mobile easy payment service factors, the relationship between the factors affecting UI relationships were explicitly and implicitly linear. However, in this study, a novel rationale was applied using a non-linear relationship analysis method. It was empirically found that the relationship between factors affecting mobile easy payment services’ UI is non-linear.

Second, it was confirmed that the risk perception of an infectious disease, such as COVID-19, is a factor that significantly affects a mobile easy payment service’s UI in the context of a pandemic. Several fields, such as tourism, education, and sports, also suggest that the prevalence of infectious diseases significantly affects consumer behavior intention. In this study too, the risk perception of health, including COVID-19, was identified as significantly affecting UI.

Third, from empirical analysis of the questionnaire items on the SN, it was found that media such as television and the internet had the largest effect on UI. Given the social distancing rules enforced due to COVID-19, face-to-face exchanges were limited, and these were the channels used to source information. Therefore, it is important for fintech companies that provide mobile easy payment services to implement marketing strategies using multiple channels. The more people perceive the service as convenient, the more they shall recognize its usefulness. Advertising this convenience shall encourage consumer usage.

Fourth, the group with a high C19RP used the mobile easy payment service to a certain level, even though its perceived usefulness was low. The group with a low C19RP used the mobile easy payment service only when they perceived its usefulness. Therefore, companies that provide mobile easy payment services should enhance its usefulness. Efforts should be made to develop various content and services, and establish a specific marketing strategy based on these results.

Despite its theoretical and practical contributions, this study has certain limitations. First, the study’s generalizability is a challenge because the participants were young (i.e., people in their teenage, 20 s, and 30 s). Given the social distancing norms, the survey was primarily conducted online with participants who were familiar with the online settings. Therefore, future research should focus on generalizing results by using samples that include diverse age groups.

Second, in the context of the ongoing COVID-19 outbreak, the study could conduct its survey for a relatively short period of 2 weeks, that is, from September 27 to October 10, 2021. This timing was dependent on the extent of COVID-19’s prevalence; therefore, there could be variances in the results. For example, the study’s survey was conducted during winter when the virus was prevalent and variants had emerged. Therefore, future studies may require a longer and/or different time span.

Third, although this study established a research model on the basis of TAM2, it is relatively simple when compared with other studies on the intention to use MEPS, which also employ TAM2. Therefore, it shall be necessary for future research to refine the study’s objectives by adding other external factor variables that possibly affect mobile easy payment services’ UI. It shall then be possible to provide comprehensive theoretical and practical implications for research on mobile easy payment services.

## Data Availability Statement

The raw data supporting the conclusions of this article will be made available by the authors upon request.

## Ethics Statement

Ethical review and approval was not required for the study on human participants in accordance with the local legislation and institutional requirements. Written informed consent for participation was not required for this study in accordance with the national legislation and the institutional requirements.

## Author Contributions

JK collected and analyzed the data. MK reviewed the final draft. Both authors designed the research study, performed the research, drafted the manuscript, contributed to the investigation, analyzed the data, read, and approved the final manuscript.

## Conflict of Interest

The authors declare that the research was conducted in the absence of any commercial or financial relationships that could be construed as a potential conflict of interest.

## Publisher’s Note

All claims expressed in this article are solely those of the authors and do not necessarily represent those of their affiliated organizations, or those of the publisher, the editors and the reviewers. Any product that may be evaluated in this article, or claim that may be made by its manufacturer, is not guaranteed or endorsed by the publisher.

## Supplementary Material

In addition, the online survey was also conducted at (https://docs.google.com/forms/d/e/1FAIpQLSddx8CN6IvP5GLucfRx_tn_NwrSKr5XHm_RaRLVmbf52nkomw/viewform?usp=sf_link).
