# Supplementary material for: Intention to Use Mobile Easy Payment Services: Focusing on the Risk Perception of COVID-19
Source: Front Psychol. 2022 May 23;13:878514. doi: 10.3389/fpsyg.2022.878514 (PMC9169679; doi:10.3389/fpsyg.2022.878514)
Supplement: Supplementary file 1 [file Data_Sheet_1.pdf]

■ 코로나19 이후 핀테크 모바일 간편결제서비스 이용 행동 의도에 영향을 미치는 요인 ■

안녕하십니까?
바쁘신 가운데 귀중한 시간 허락해 주셔서 감사합니다.
본 설문지는 '코로나19이후 핀테크 모바일 간편결제서비스 이용 행동 의도에 영향을 미치는 요인'를 위한 설문지입니다. 응답내용은 통계법 제33조(비밀의 보호) 및 제34조 통계종사자 등의 의무)에 의해 비밀이 절대 보장되며, 통계목적 이외에는 절대 사용되지 않습니다.
바쁘시더라도 잠시 시간 내어 답변 부탁드립니다.

2021. 09

* 연구자 : 제주대학교 일반대학원 경영정보학과 김지원

*** 설문을 진행하는 것에 대해 동의하는 경우, 동의에 체크(√) 하신 후 설문을 진행해주시기 바랍니다.**

(비동의 체크시 설문 진행을 종료해주십시오.)

1. 동의 ☐　　　　2. 비동의 ☐

*** 모바일 간편결제 서비스**

: 카드 정보를 모바일기기 등에 미리 저장해 두고 오프라인이나 온라인 상에서 소비자가 물건이나 서비스를 구매하려고 할 때 비밀번호 입력, 단말기 접촉 등의 방법으로 결제하는 서비스

ex) 삼성페이, 네이버페이, 카카오페이 등

**<삼성페이>**	**<네이버페이>**	**<카카오페이>**

Ⅰ. 귀하께서는 국내 COVID-19 발병 기간(2020년 1월 20일~ 현재) 동안 모바일 간편결제 서비스를 이용해본 경험이 있으십니까?

① 있음 ② 없음

Ⅱ. 아래 항목들에 대해 귀하께서 해당하는 정도에 표시하여 주십시오.

항 목	전혀 그렇지 않다	그렇지 않다	그저 그렇다	그렇다	매우 그렇다
1. 내게 영향을 미치는 사람들(ex. 가족, 친구, 직장동료 등)은 내가 모바일 간편결제서비스를 이용하는 것을 동의한다(또는 동의할 것이다).	①	②	③	④	⑤
2. 나를 중요하게 생각하는 사람들은 내가 모바일 간편결제서비스를 이용하는 것을 긍정적으로 생각한다(또는 생각할 것이다).	①	②	③	④	⑤
3. 내 주변 사람들은 나에게 모바일 간편결제 서비스를 이용해 보라고 권하는 사람이 많다(또	①	②	③	④	⑤

는 많을 것이다).					
4. 나와 가까운 사람들은 내가 모바일 간편결제 서비스를 이용하는 것을 찬성한다(또는 찬성할 것이다).	①	②	③	④	⑤
5. 나는 신문, 방송, 인터넷 매체를 보면 모바일 간편결제 서비스를 이용해봐야겠다는 생각이 든다(생각이 들 것이다).	①	②	③	④	⑤

Ⅲ. 아래 항목들에 대해 귀하께서 해당하는 정도에 표시하여 주십시오.

항 목	전혀 그렇지 않다	그렇지 않다	그저 그렇다	그렇다	매우 그렇다
1. 나는 모바일 간편결제서비스를 통한 결제가 쉽다(또는 쉬울 것이다).	①	②	③	④	⑤
2. 나는 모바일 간편결제서비스를 사용하는 것이 쉽다(또는 쉬울 것이다).	①	②	③	④	⑤
3. 나는 모바일 간편결제서비스 사용법을 익히는 것이 쉽다(또는 쉬울 것이다).	①	②	③	④	⑤
4. 나는 모바일 간편결제서비스를 능숙하게 사용한다(또는 사용할 것이다).	①	②	③	④	⑤
5. 나는 모바일 간편결제서비스를 통해 결제하는 것이 어렵지 않다(어렵지 않을 것이다).	①	②	③	④	⑤

Ⅴ. 아래 항목들에 대해 귀하께서 해당하는 정도에 표시하여 주십시오.

항 목	전혀 그렇지 않다	그렇지 않다	그저 그렇다	그렇다	매우 그렇다
1. 모바일 간편결제서비스를 이용하면 더 빨리 결제할 수 있다(또는 있을 것이다).	①	②	③	④	⑤
2. 모바일 간편결제서비스를 이용하면 결제가 더 쉬워진다(또는 쉬워질 것이다).	①	②	③	④	⑤
3. 기존 결제수단(현금/카드)보다 모바일 간편결제서비스가 더 유용하다(또는 유용할 것이다).	①	②	③	④	⑤

4.	모바일 간편결제서비스가 앞으로 더 유용해진다(또는 유용해질 것이다).	①	②	③	④	⑤
5.	모바일 간편결제서비스가 단점보다 장점이 더 많다고 생각한다(또는 많을 것이라고 생각한다).	①	②	③	④	⑤

Ⅵ. 아래 항목들에 대해 귀하께서 해당하는 정도에 표시하여 주십시오.

항 목		전혀 그렇지 않다	그렇지 않다	그저 그렇다	그렇다	매우 그렇다
1.	현금이나 카드 결제수단을 이용하면 확진자와 접촉할 수 있다.	①	②	③	④	⑤
2.	현금이나 카드 결제수단을 이용하면 코로나19에 감염될 수 있다.	①	②	③	④	⑤
3.	현금이나 카드 결제수단을 이용하면 모바일 간편결제서비스보다 코로나19 바이러스에 더 노출된다.	①	②	③	④	⑤
4.	현금이나 카드결제 수단을 이용하면 모바일 간편결제보다 코로나19 바이러스에 감염을 증가시킨다.	①	②	③	④	⑤
5.	현금이나 카드결제 수단 이용하면 거스름돈이나 카드에 코로나19 바이러스 비말이 남아 있을 수 있다.	①	②	③	④	⑤

Ⅶ. 아래 항목들에 대해 귀하께서 해당하는 정도에 표시하여 주십시오.

항 목		전혀 그렇지 않다	그렇지 않다	그저 그렇다	그렇다	매우 그렇다
1.	나는 일상생활에서 모바일 간편결제서비스를 사용할 것이다(또는 계속 사용할 것이다).	①	②	③	④	⑤
2.	나는 모바일 간편결제서비스를 사용할 의향이 있다(또는 계속 사용할 의향이 있다).	①	②	③	④	⑤
3.	나는 모바일 간편결제서비스를 주변인에게 긍정적으로 평가하고 추천할 것이다.	①	②	③	④	⑤

4.	나는 생활능률을 높이기 위해 모바일 간편결제서비스를 사용할 것이다(또는 계속 사용할 것이다).	①	②	③	④	⑤
5.	나는 결제수단으로 모바일 간편결제서비스를 이용할 것이다(또는 계속 이용할 것이다).	①	②	③	④	⑤

Ⅷ. 일반사항

1. 성 별	① 남		② 여			
2. 연 령	① 20대 미만	② 20대	③ 30대	④ 40대	⑤ 50대 이상	
3. 직 업	① 농업, 어업, 임업	② 자영업	③ 기술직	④ 사무직	⑤ 전문직	
	⑥ 서비스직	⑦ 연구직	⑧ 학생	⑨ 주부	⑩ 무직(또는 퇴직)	⑪ 기타
4. 학 력	① 중학교 졸업이하 (재학 포함)	② 고등학교 졸업 (재학 포함)	③ 전문대학 졸업 (재학 포함)	④ 대학교 졸업 (재학 포함)	⑤ 대학원 이상 (재학 포함)	

설문에 응해주셔서 대단히 감사합니다.
